# Resilience and relapse risk in Emirate adult patients with substance use disorder: a national cross-sectional study from the United Arab Emirates

**DOI:** 10.3389/fpsyt.2024.1444233

**Published:** 2024-09-24

**Authors:** Ibraheem Mhaidat, Nabeel Al-Yateem, Samya Al-Mamari, Fatima Al-Suwaidi

**Affiliations:** ^1^ Department of Nursing, The National Rehabilitation Center, Abu-Dhabi, United Arab Emirates; ^2^ Colleague of Health Science, University of Sharjah, Sharjah, United Arab Emirates; ^3^ Medical Sector, The National Rehabilitation Center, Abu-Dhabi, United Arab Emirates

**Keywords:** resilience, relapse, mental health, substance use disorder, substance misuse

## Abstract

**Introduction:**

**The** United Arab Emirates is among the countries affected by substance use disorders (SUDs), which have economic and social impacts. Relapse after successful rehabilitation is a major issue in the treatment of SUD. Several factors increase the risk of relapse in patients with SUD, such as craving and negative social experiences. Resilience could empower patients struggling with SUD. This study aims to explore levels of resilience and relapse risk in adult Emirati patients diagnosed with SUD, and also the possible correlation between the two variables.

**Methods:**

Two hundred eighty-six Emirati adult patients with SUD completed a self-administered questionnaire for demographics, resilience (Connor and Davidson Resilience Scale), and relapse risk (Stimulant Relapse Risk Scale). Descriptive statistics (frequency, percentage, mean, SD, etc.) were used to describe study participants and variables. Inferential statistics were used to analyze the relationships, associations, and correlations between resilience and relapse risk, the main variables, and the participants demographics.

**Result:**

Participants’ mean total score for resilience was 72.92 out of a maximum possible score of 100 (SD = 16.99), while their mean total score for relapse risk was 59.07 out of a maximum possible score of 105 (SD = 12.23). Furthermore, examining the correlation between the resilience subscales and the relapse risk subscales revealed similar significant, negative, low-to-moderate correlations between all the subscales (r = -0.486).

**Discussion:**

Protective and risk factors to enhance resilience and reduce relapse risk in patients with SUD were discussed.

## 1 Introduction

The prevalence of Substance Use Disorder (SUD) continues to rise globally (1). During the early ‘80s, the United Arab Emirates (UAE) began experiencing the spread of this disorder, notably affecting nationals more than expatriates (2). A study found that 48.2% of Emirati patients discharged between January 1, 2015, and December 31, 2015, from a psychiatric hospital in Abu Dhabi city had a SUD diagnosis upon discharge (3). This phenomenon was attributed to factors such as westernization, globalization, the influx of a massive multinational workforce (2), geographical location facilitating drug trading, rapid population growth, shifts in social norms (4, 5), and the transition from poverty to wealth (6). The detrimental effects of SUD in the UAE have attracted significant attention, with over eight thousand people detained for drug-related crimes in 2021 (7). Furthermore, the economic impact of addiction in the UAE was estimated at $5.47 billion, or 1.4% of GDP, in 2012 (8).

## 2 Literature review

### 2.1 Relapse

It is well known that SUD is refractory to treatment. In the USA, 51% of patients experienced a relapse to substance use following discharge from residential treatment (9). Approximately, the same number relapsed during the first six months of outpatient treatment, and more than 32% dropped out during that period in USA (10). In Rwanda, a study found that 59.9% of the participants experienced one or more relapses after being discharged (11). Patients with alcohol use disorder (AUD) experience higher relapse rates, with 68% to 73% relapsing following treatment (12, 13). A study in the UAE reported that out of the 368 patients admitted to the National Rehabilitation Center (NRC) in Abu Dhabi between 2002 and 2011, 27.2% were readmitted at least once (14). However, the UAE’s relapse rate is anticipated to be higher, given that it is calculated by tracking patients over time post-successful rehabilitation.

Factors contributing to the high risk of relapse include: craving and the body’s counter-reaction to the substance used (15, 16), which can vary depending on the specific drug, living in low socioeconomic status areas, living alone (17), sleep disruption (18), living with only their mothers, spending less than three months in the rehabilitation program, using more than one substance, living with peers or drug dealers, family-related problems (11), negative social experiences (9), comorbid mental illness (19, 20), and genetic composition (i.e., Met/Met gene) (12). Conversely, factors such as insight and social support (21) and employment (22) showed a reduction in the risk of relapse. The UAE is a welfare state dedicated to providing support, including employment and housing, to its citizens. Nonetheless, patient resistance and social stigma impede the participation of Emirati patients in SUD treatment programs (23). In-depth studies on SUD in the UAE are limited (4, 24). To the best of our knowledge, there have been no studies examining the risk of relapse in adult Emirati patients diagnosed with SUD.

### 2.2 Resilience

Empowering mental health patients themselves is an important strategy in the UAE and the general Arabic culture. A concept that has recently garnered significant attention is resilience, which is both intriguing and valuable. Resilience can be defined as “a process whereby people bounce back from adversity through the protective factors that serve to moderate the effects of adversity” (25) or successful stress-coping ability (26). Resilience is an incredible process with the potential to enhance various aspects of human life, including toughening affect and increased coping abilities (25), elevated self-efficacy (27), greater life satisfaction (28, 29), improved self-esteem and social self-efficacy (30), and enhanced spiritual intelligence (22). The encouraging news is that resilience is modifiable process. In one study, a combination of “acknowledging thoughts and emotions” and “breathing exercises” was employed to show improved resilience among adults (31).

The building blocks of resilience consist of both protective factors and life adversities. The literature has identified numerous protective factors that contribute to resilience. For instance, protective factors such as strong connections with competent and caring adults within the family and community, cognitive and self-regulation skills, positive self-perception, and motivation contribute to the development of highly resilient children, ultimately enhancing their growth (32). Self-acceptance, self-care strategies, peer support, altruism, and access to resources like insurance serve as protective factors that have the potential to enhance resilience in women with opioid use disorder (33).

To understand resilience within a specific group, it’s essential to identify the unique protective factors and adversities that define resilience in that context. This understanding, in turn, enhances resilience. According to Masten (2001), protective factors vary based on cultural norms (32). In the context of SUD, protective factors can be identified by examining accessible healthy skills and abilities and recognizing adversities (25).

### 2.3 Resilience and relapse in SUD

Previous studies, though conducted within different context, showed that resilience is associated with a reduced risk of relapse (22), by fostering greater awareness and mindfulness toward the progress within recovery capital domains (34). Literature showed a range of protective factors that underlie improvement in relapse in patients with SUD. For instance, being able to feel both positive and negative emotions at the same time during a high-stress situation can enhance flexible thinking and problem-solving abilities, and help protect individuals from the negative effects of stress, including relapse in patients with SUD (35). Similarly, optimism improves the stress response in patients with AUD (36). Another characteristic of resilient individuals, having high social contacts, has been associated with lower alcohol use (37). Another study showed that providing family members with various resources to assist them in completing tasks reduces relapse by enhancing resilience and self-esteem, as resilience is improved through the alleviation of negative emotional states associated with poor family functioning (38). In periods of stress, the spiritual aspect of resilient patients can provide meaning in stressful situations, aiding them in overcoming relapse when they need assistance in dealing with life’s pressures (39). Furthermore, cognitive reappraisal skills, which target factors including cognitive flexibility and negative thought patterns known to be affected in patients with SUD, improve resilience and result in enhanced social support, further protecting against relapse (37).

In the UAE, there are several qualitative studies on Emirate patients with SUD that identified some protective factors, such as religiosity, and adversities, such as insufficient treatment, that could affect treatment outcomes (23, 40, 41). Resilience, however, is distinct in that it arises from the interplay between these protective factors, vulnerabilities, and the experienced adversities, shaping human outcomes (25, 33, 42). Studying and measuring resilience can help us comprehend these interactions (43), yet such research has not been conducted among Emirate adult patients with SUD. It’s essential to note that individuals with high resilience are expected to perform well in specific aspects of life, not necessarily all (44). Luthar et al. argued that specifying resilience outcomes is essential for determining the protective factors and adversities that interact to shape those outcomes (44). In our case, the outcome will be relapse risk in adult Emirati patients with SUD.

This study aims to explore levels of resilience and relapse risk in adult Emirati patients diagnosed with SUD. as well as the possible correlation between the two variables. Specifically, the study will attempt to answer the following main questions:

• Is there a relationship between resilience and relapse risk in adult Emirati patients diagnosed with substance use disorder?

• Is there a difference in relapse risk scores according to relapse status?

• What are the resilience and relapse risk scores for Emirate adult patients with SUD?

• Is there a difference in resilience and relapse risk scores among the sample subgroups (i.e., different genders, educational backgrounds, employment status, etc.)?

## 3 Methods

### 3.1 Study design

A cross-sectional correlational survey design was employed to assess relapse risk and resilience and to explore the relationship between these variables among patients with SUD

### 3.2 Data collection

Questionnaires were completed by both inpatient and outpatient individuals at the NRC. The NRC serves as the primary national referral center for Emirati patients diagnosed with SUD from across the UAE. It encompasses seven buildings covering a total area of 108,000 square meters. The facility offers 169 inpatient beds, accommodating both men and women, and provides a range of treatment options tailored to various stages of recovery. Additionally, the NRC includes facilities for medical services, men’s assessments, outpatient care, a mixed-use building, and a central block building.

There are more than six thousand Emirati patients who have received treatment for SUD at the NRC and are now managing their condition at home. Some of them regularly return to the Outpatient Departments (OPD) for routine check-ups. Approximately one hundred patients are seen weekly in the OPD. Since there is no available data on the prevalence of SUD among the Emirati population, consecutive sampling was employed. This approach was chosen because the known and accessible population consists of those available at the NRC, which serves as the primary rehabilitation center specialized in the treatment of SUD in the UAE.

All outpatients and those who were admitted to the NRC between June 1, 2022, and the end of March 2023, were invited to participate in the study. This invitation involved approaching each patient individually, explaining the study, and obtaining their consent. Additionally, all patients attending the OPD during the same period with a confirmed diagnosis of SUD were invited to participate. This invitation was extended through posters displayed at the main entrance and by approaching each patient individually. Approximately 40% of those invited participated in the study. A list of the file numbers of participating patients was maintained to prevent duplication since patients have follow-up appointments scheduled weekly, every two weeks, or every three weeks.

Any patient who agreed to participate was escorted to a meeting room where the study was thoroughly explained. Both verbal and written consent forms containing all the necessary study information were obtained. The principal author of this study personally explained these forms to the participants. Each participant was assured that they could withdraw from the study at any time without any adverse consequences. To ensure participants’ confidentiality, all questionnaires were assigned numerical codes before being provided to the subjects.

The principal author remained present with the patients to address any questions or concerns they might have. After completing the questionnaire, it was reviewed for any missing data and then returned to the patient for their completion.

### 3.4 Measurement

#### 3.4.1 The translation procedure

The resilience and relapse risk scales were translated by two bilingual mental health professionals from English to Arabic. Any discrepancies were resolved through consensus and subsequently edited by a professional translator, resulting in Version 1. Next, Version 1 was back-translated by two different bilingual mental health professionals who were not familiar with the original text, generating Version 2. Both versions were then reviewed by two different mental health professionals and a second professional translator, and any discrepancies were addressed to ensure language equity.

The comprehensibility and readability of the translated questionnaires were assessed during a preliminary pilot study that recruited 25 participants visiting or admitted to the NRC for treatment. A content validity questionnaire was created, encompassing all items that would constitute the final questionnaire. The Cronbach’s alpha for the translated version of the resilience scale was 0.904, indicating high reliability. Similarly, the Cronbach’s alpha for the translated version of the relapse risk scale was 0.901, also indicating high reliability.

#### 3.4.2 Resilience

The English version of the Connor-Davidson Resilience Scale (CD-RISC25) (26) was translated to Arabic through the translation procedure and was used to measure resilience in patients with SUD. The CD-RISC25 consists of twenty-five items measured on a Likert scale with the following responses: not true at all (0), rarely true (1), sometimes true (2), often true (3), and true nearly all of the time (4). Scores range from 0 to 100, with higher scores indicating greater resilience.

#### 3.4.3 Relapse risk

The English version of the Stimulant Relapse Risk Scale (SRRS) (45) was translated to Arabic through the translation procedure and was used to measure the risk of relapse. Previous Studies employed SRRS to predict relapse in patients who had used stimulants and other drugs (22, 46). The SRRS comprises 35 items measured on a Likert scale with the following responses: strongly disagree and disagree (1), neither disagree nor agree (2), agree and strongly agree (3). Higher scores indicate a greater risk of relapse.

#### 3.4.4 Demographics

Demographic variables for patients with SUD include gender, age, monthly income, marital status, education, city, employment status, living arrangements (alone or with a supportive one), family history of SUD, age at drug initiation, age at treatment initiation, time since last drug use, types of drugs used, presence of physical disorders, presence of psychological disorders, the unit of admission, cumulative time spent in rehabilitation programs, and cumulative time spent in OPD.

Urine and alcohol tests were collected to classify participants as relapsed, lapsed, abstinent, or for new assessments by the treating physician on the same day as data collection. A relapse is a return to substance use after a period of abstinence, often signaling a breakdown in recovery, while a lapse is a brief return to substance use following a period of abstinence; unlike a relapse, which can involve a more prolonged or sustained return to substance use, a lapse is typically a single or short-term event, and abstinent refers to refraining from substance use entirely (47). Undergoing a new assessment means the patient’s first exposure to treatment for SUD, involving an initial evaluation of their condition and needs. The treating physician was also responsible for confirming the diagnosis of SUD and any other psychiatric comorbidities.

### 3.5 Data analysis

Descriptive statistics (Frequencies, Percentages, Mean, SD, etc.) were used to describe the study participants and variables. Reliability and validity statistics for all data collection tools were calculated including Cronbach’s alpha coefficients, item-to-item correlations, item-to-scale correlations, and construct validity. Inferential statistics were employed to analyze the relationships, associations, and correlations between resilience and relapse risk, which are the main variables, as well as the demographic characteristics of the participants.

## 4 Result

### 4.1 Resilience and relapse risk scales reliability and validity test

The resilience scale was evaluated for reliability and validity. The Cronbach’s alpha coefficient was found to be 0.904, indicating a high level of reliability. Additionally, item-to-item correlations, item-to-scale correlations, and Cronbach’s alpha for the scale were assessed to determine item relationships. All items exhibited positive correlations with each other, and each item also displayed a positive correlation with the total scale, with correlation coefficients ranging from 0.2 to 0.6. Importantly, the Cronbach’s alpha for the entire scale remained consistent when any item was removed.

The relapse scale was also evaluated for reliability and validity. The Cronbach’s alpha coefficient was found to be 0.901, indicating a high level of reliability. Additionally, item-to-item correlations, item-to-scale correlations, and Cronbach’s Alpha for the scale were assessed to determine item relationships. All items exhibited positive correlations with each other, and each item also displayed a positive correlation with the total scale, with correlation coefficients ranging from 0.16 to 0.69. Importantly, the Cronbach’s alpha for the entire scale remained consistent when any item was removed.

### 4.2 Demographics

Participants’ ages were distributed across several categories, with the largest group being those aged 29–34 years (34.6%), followed by participants aged 24–28 years (26.6%). The majority of participants were male (95.5%) and lived with their families (94.4%). Most participants reported having no personal monthly income (42.0%), followed by those with incomes in the range of ‘10,001–20,000 AED’ (26.9%). A significant proportion of participants were not married (55.2%), and in terms of employment, the largest group was those not working (50.3%), followed by those employed in government jobs (40.2%). The majority of participants had completed secondary school (52.8%), with smaller percentages having completed bachelor’s degrees (18.2%). Abu Dhabi was the most common city of residence (60.5%).

Regarding participants’ medical histories, most reported no history of medical co-morbidities such as hypertension, diabetes, hepatitis, or HIV. However, a significant percentage reported a history of psychological co-morbidities (36.7%).

The mean age at which participants began using drugs was 19 years, with the onset of drug use ranging from 11 to 51 years old. Forty-nine percent of participants started using drugs before the age of 18, and 61% began consumption at or before the age of 18. The mean age at which they began treatment was 27 years, resulting in an average gap of 8 years between drug use initiation and treatment initiation. The time since the last drug use ranged from 1 to 1825 days. **Table 1** provides a detailed overview of the participants’ demographic characteristics. The majority of the participants (66.4%) reported using four or more substances, while a smaller proportion reported using two to three substances (20.3%) or one substance (13.3%).

**Table 1:** Details of the participants demographic characteristics (N = 286).

Items	Subcategory	Count	N %
**Number of participants**		286	
**Age Group**	18-28	113	40%
	29-40	140	49%
	41-51	25	9%
	Above 51	8	3%
**Gender**	Male	273	96%
	Female	13	5%
**Monthly income**	No income	120	42%
	Less than 10,000	32	11%
	10,001-20,000	77	27%
	20,001-30,000	30	11%
	30,001-40,000	14	5%
	Above 40,000	13	5%
**Marriage status**	Single	158	55%
	Married	99	35%
	Divorced	27	9%
	Widow	2	1%
**Do you live alone or with family**	Alone	16	6%
	With family	270	94%
**Employment**	Governmental	115	40%
	Private	17	6%
	Charity	3	1%
	Business	7	2%
	Not working	144	50%
**Education**	School education only	211	74%
	Diploma	15	5%
	Bachelor	52	18%
	Master or PhD	8	3%
**City**	Abu Dhabi	173	61%
	Dubai	36	13%
	Sharjah	33	12%
	Ajman	16	6%
	Umm Alquwain	3	1%
	Ras Al Khaimah	15	5%
	Al-Fujairah	10	4%
**Family History of SUD**	No	201	70%
	Yes	85	29.7%
**Medical Co-Morbidities (HTN, DM, Hepatitis)**	No	244	85%
	Yes	42	15%
**HIV History**	No	286	100.0%
	Yes	0	0.0%
**Psychological comorbidities**	No	181	63.3%
	Yes	105	36.7%
**Age when started drug use**	Mean (M)	19	
	Standard Deviation (SD)	7	
**Age when started treatment**	Mean (M)	27	
	Standard Deviation (SD)	8	
**Time since last drug use (in days)**	Mean (M)	260	
	Standard Deviation (SD)	38.2	
**Number of admissions**	None	53	18.5%
	Once	133	46.5%
	Twice	50	17.5%
	Thrice	23	8.0%
	Four times	13	4.5%
	Five times	4	1.4%
	Above the mentioned	10	3.5%
**Cumulative admission time (in months)**	Mean (M)	2.996	
	Standard Deviation (SD)	4.224	
**Cumulative OPD time (in months)**	Mean (M)	10.225	
	Standard Deviation (SD)	17.993	
**Total OPD discrete treatment episodes**	Mean (M)	17.163	
	Standard Deviation (SD)	28.49	
	Minimum (Min)	1	
	Maximum (Max)	216	
**Relapse Status**	New Assessment	75	26.2%
	Relapse	91	31.8%
	Lapse	18	6.3%
	Abstinent	102	35.7%

The most frequently used substance among participants was Crystal (77%, n = 220), followed by THC (65%, n = 187), Lyrica (58%, n = 166), Tramadol (52%, n = 150), and Morphine (50%, n = 144). All of these substances were used by more than half of the participants. A detailed list of the substances used is provided in **Table 2**.

**Table 2:** List of the substances used.

Substance	Frequency	%
**Crystal**	220	76.90%
**THC**	187	65.40%
**Lyrica**	166	58.00%
**Tramadol**	150	52.40%
**Morphine**	144	50.30%
**Alcohol**	131	45.80%
**Heroin**	123	43.00%
**Xanax**	109	38.10%
**Captagon**	100	35.00%
**Spice**	97	33.90%
**Valium**	96	33.60%
**Marijuana**	86	30.10%
**Kemadrin**	82	28.70%
**Cocaine**	62	21.70%
**Artane**	45	15.70%
**GGT**	40	14.00%
**Al-Khat**	21	7.30%
**Total Number of** **Substances Used**	**Frequency**	**Percent**
**One substance**	38	13.3%
**Two to three substances**	58	20.3%
**Four or more substances**	190	66.4%

**Figure 1:**
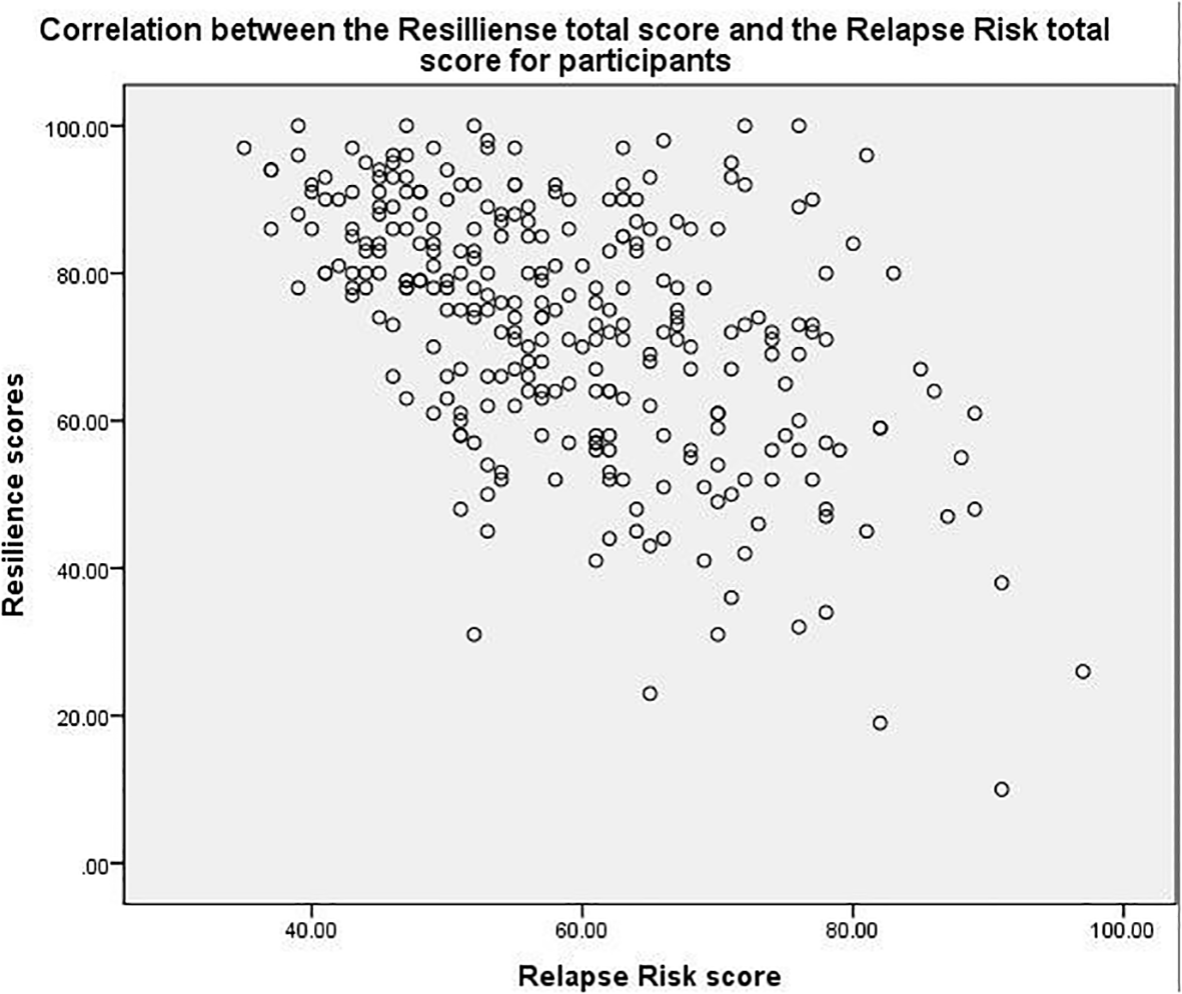
Correlation between resilience and relapse risk.

The majority of the participants were abstinent (35.7%), followed by those who experienced relapse (31.8%), new assessments (26.2%), and lapses (6.3%). Most participants were admitted once (46.5%), followed by those with no admissions (18.5%), twice (17.5%), thrice (8.0%), four times (4.5%), more than five times (3.5%), and five times (1.4%). The average cumulative admission time was approximately 2.996 months, with a standard deviation of 4.224 months. The average cumulative OPD time was approximately 10.225 months, with a standard deviation of 17.993 months. The average number of OPD discrete treatment episodes was 17.163, with a standard deviation of 28.49, ranging from a minimum of 1 to a maximum of 216 episodes. **Table 1** provides a detailed overview of the participants’ demographic characteristics. Participants mean total score for resilience was 72.92 out of a maximum possible score of 100 (SD = 16.99), while their mean total score for relapse risk was 59.07 out of a maximum possible score of 105 (SD = 12.23). When examining the correlation between resilience and relapse risk, a significant, negative, and moderate correlation with a significance level of less than 0.001 and a correlation coefficient of -0.486. **Figure 1** illustrates this correlation. Additionally, when examining the correlation between the resilience subscales and the relapse risk subscales similar significant, negative, low-to-moderate correlations were observed between all the subscales. Details of the subscales for resilience and relapse risk are provided in **Tables 3A, B**. 

**Table 3:** Inferential statistics results.

TABLE 3A Details of the resilience and relapse risk scales.					
Subscale	Minimum	Maximum	Mean	SD	
**Resilience Subscale 1**	2	32	24.6783	6.39803	
**Resilience Subscale 2**	3	28	17.9615	5.66695	
**Resilience Subscale 3**	0	20	14.7587	4.10492	
**Resilience Subscale 4**	0	12	8.6713	2.91584	
**Resilience Subscale 5**	1	8	6.8531	1.59366	
**Resilience Total**	10	100	72.9231	16.9875	
**Relapse Subscale 1**	8	24	13.549	3.51678	
**Relapse Subscale 2**	8	24	15.8601	3.66319	
**Relapse Subscale 3**	4	12	4.9406	1.63942	
**Relapse Subscale 4**	6	18	10.1923	3.62565	
**Relapse Subscale 5**	4	11	6.5664	1.70473	
**Relapse Subscale 6**	5	15	7.9615	2.28233	
**Relapse Total**	35	97	59.0699	12.2278	
Table 3B Details of the resilience and relapse risk sub scales correlations.					
Subscale	Resilience Sub1	Resilience Sub2	Resilience Sub3	Resilience Sub4	Resilience Sub5
**Relapse Sub1**	-0.380*	-0.294*	-0.284*	-0.408*	-0.157*
**Relapse Sub2**	-0.457*	-0.299*	-0.385*	-0.421*	-0.176*
**Relapse Sub3**	-0.288*	-0.237*	-0.259*	-0.275*	-0.161*
**Relapse Sub4**	-0.374*	-0.272*	-0.344*	-0.373*	-0.159*
**Relapse Sub5**	-0.061	-0.016	-0.127*	-0.113	-0.075
**Relapse Sub6**	-0.336*	-0.281*	-0.295*	-0.327*	-0.104

Signifies that the p-value is less than 0.05.

### 4.3 Resilience & relapse risk for patients who are: abstinent, relapse, lapse, new assessment

The resilience and relapse risk scores of participants classified as abstinent, relapsed, lapsed, or undergoing a new assessment were also assessed. Resilience scores for all these participants were similar, with no significant differences between their scores. However, the relapse risk scores for these participants were significantly different. Scores of participants who experienced lapses or relapses were significantly higher than the scores of other participants classified as new assessments or completely abstinent (p < 0.001).

### 4.4 Resilience & relapse risk and psychological comorbidities

When assessing the difference between the resilience and relapse risk scores for participants with other psychological comorbidities, a significant difference was found. Generally, participants with psychological comorbidities had lower resilience scores, both in the total score and in the subscales. Similarly, the relapse risk scores for the two groups were significantly different. The mean relapse risk score was higher in participants with psychological comorbidities, both in the total score and in the subscales.

The impact of medical comorbidities such as HTN, DM, and Hepatitis on the resilience and relapse risk scores was also assessed. The findings revealed no statistically significant differences in scores between participants with and without these disorders. However, having a family member who had used illegal drugs before was significantly associated with a higher risk of relapse among the participants (p = 0.01). Statistically, this did not have an effect on the resilience scores of the participants (p = 0.365).

### 4.5 Resilience & relapse risk and substances used

There was no significant difference in resilience scores between the groups based on the type or the number of substances used. However, the Mann-Whitney U tests revealed significant differences in relapse risk based on the type of substance used. Significant differences were observed for Valium (p = 0.004), Morphine (p = 0.002), Heroin (p < 0.001), Tramal (p = 0.001), Xanax (p = 0.001), Lyrica (p = 0.001), and Spice (p = 0.006). Other substances did not show significant differences in relapse risk. The Kruskal-Wallis test also indicated a significant difference in relapse risk based on the number of substances used, with those using four or more substances having a higher relapse risk (Mean Rank = 158.95) compared to those using two or three substances (Mean Rank = 126.94) and one substance (Mean Rank = 91.54) (p < 0.001).

### 4.6 Correlation between admission time and relapse and resilience and OPD time and relapse and resilience

The correlations between the cumulative admission time for participants and their relapse risk and resilience scores were assessed. The correlation of cumulative admission time was not significant for both relapse risk and resilience scores. On the other hand, the correlations between the cumulative treatment time spent in the OPD for participants and their relapse risk and resilience scores revealed that the cumulative OPD time correlated significantly, positively, and weakly with the resilience score. This indicates that individuals with higher resilience spent more time at the OPD clinics.

In terms of age, education, and income, the analysis revealed no significant differences between participants in different categories of these variables concerning their resilience and relapse risk scores. However, significant differences were observed in resilience and relapse risk among categories of the employment variable. Individuals who were not working showed lower resilience scores and higher relapse risk in comparison to those who are working. Detailed inferential statistics results are provided in **Tables 4A**, **B**.

**Table 4A:** Resilience and relapse risk comparisons (N = 286).

Test	Variable	Count	U or H Statistic	Z or Degrees of Freedom	Mean Rank	Significance (p-value)
Mann-Whitney U Test: Resilience Comparisons						
	Psychological Comorbidities	Yes: 105 No: 181	6305.5	-4.743	Yes: 113.05 No: 161.16	<0.001*
	Employment	Yes: 142 No: 144	8261.5	-2.807	Yes: 157.32 No: 129.87	0.005*
Mann-Whitney U Test: Relapse Risk Comparisons						
	SUD Family History	Yes: 85 No: 201	6900	-2.570	Yes: 162.82 No: 135.33	0.01*
	Psychological Comorbidities	Yes: 105 No: 181	5564	-5.844	Yes: 181.01 No: 121.74	<0.001*
	Employment	Yes: 142 No: 144	7875.5	-3.359	Yes: 126.96 No: 159.81	.001*
	Valium	Yes: 96 No: 190	7208.5	-2.895	Yes: 163.41 No: 133.44	0.004*
	Morphine	Yes: 144 No: 142	8080	-3.067	Yes: 158.39 No: 128.4	0.002*
	Heroin	Yes: 123 No: 163	7247	-4.013	Yes: 166.08 No: 126.46	<0.001*
	Tramal	Yes: 150 No: 136	7810.5	-3.422	Yes: 159.43 No: 125.93	0.001*
	Xanax	Yes: 109 No: 176	7401	-3.241	Yes: 163.1 No: 130.55	0.001*
	Lyrica	Yes: 166 No: 119	7645	-3.254	Yes: 156.45 No: 124.24	0.001*
	Spice	Yes: 97 No: 189	7364	-2.723	Yes: 162.08 No: 133.96	0.006*
Kruskal-Wallis Test: Relapse Risk Comparisons						
	Relapse Status	New: 75, Relapse: 91, Lapse: 18, Abstinent: 102	26.881	3	New: 125.41, Relapse: 177.91, Lapse: 163.19, Abstinent: 122.62	<0.001*
	Number of Substances Used	One: 38, Two or three: 58, Four or more: 190	23.97	2	One: 91.54, Two or three: 126.94, Four or more: 158.95	<0.001*

Signifies that the p-value is less than 0.05.

**Table 4B:** Spearman’s correlations.

Item	Significance	Spearman’s Coeff
**Resilience Vs relapse risk**	0.001*	-0.486
**Resilience Vs Cumulative admission**	0.578	0.033
**Resilience Vs OPD cumulative time**	0.035*	0.125
**Relapse Risk Vs Cumulative admission**	0.443	0.046
**Relapse Risk Vs OPD cumulative time**	0.846	-0.012

Signifies that the p-value is less than 0.05.

## 5 Discussion

This research holds significant value as it is among the first investigations within Arab and Islamic countries, including the UAE, to delve into the prevalence of drug misuse disorders. The subject matter, stigmatized across the region, faces a considerable deficit in both quantitative data and rigorous research. Substance use recovery involves both abstinence and recovery capital. While abstinence is praised for its positive effects on well-being and overall healthy functioning (48), research has shifted toward a more holistic view beyond just use frequency (49). By enhancing the resilience of individuals with SUDs against relapse, this study aims to equip them with the tools necessary to navigate the pressures and triggers often associated with a return to substance misuse. The ultimate goal is not only to assist individuals in managing and recovering from their SUDs but also to establish a foundation for future research and policy development in this under-explored and stigmatized domain across these nations.

The mean total score for relapse risk in our sample was also concerning. Patients with SUDs in the USA tend to experience higher relapse risk and greater psychological pathology compared to other populations. Among individuals with any prior SUDs in the USA, a small percentage were abstinent during the year preceding data collection (50). Patients who use heroin have a notably high relapse rate, as do patients who use cocaine and methamphetamine ​ (51)​. These statistics highlight the pervasive risk of relapse among individuals with SUD in the USA, driven by factors such as anxiety and disease burden (52). In contrast, Emirati patients may benefit from different socio-cultural and environmental factors that lower their relapse risk. Additionally, socioeconomic factors, healthcare access, and the role of religion differ between the two countries. Emirati patients with SUDs, despite these protective factors, still face a considerable risk of relapse.

The study findings showed that the mean total resilience score was relatively low. When compared to the general population resilience scores in the United States, Emirati adult patients diagnosed with SUDs fall within the lowest 25th percentile (26). However, comparing resilience across the USA and UAE presents several methodological challenges. In the USA, resilience often reflects individualistic values like self-reliance, whereas in the UAE, a more collectivist culture emphasizes family and community support. Standard resilience measures, designed with Western contexts in mind, may not fully capture the communal and spiritual dimensions valued in the UAE. Adversities that contribute to lower resilience mirror those highlighted in previous studies and reviews, particularly emphasizing the harmful effects of stigma on self-esteem, the disruption of family dynamics, and the challenges in emotional regulation (23). These and other adversities should be addressed through targeted interventions and support systems to enhance overall resilience.

The correlation between resilience and relapse risk revealed a significant, negative, and moderate correlation. Beyond stigma, Emirati patients with SUD encounter several other adversities that increase the risk of relapse, including unstructured time, communication breakdown within families due to unfamiliarity with the SUD disease process, decreased trust in the patient’s abilities and the family’s belief in their sincerity due to multiple failed attempts, leading to a loss of family support, continued association with friends who use drugs, legal issues, difficulties in securing employment, and inadequate treatment, such as a lack of follow-up appointments (23). Emirati patients with SUD face intellectual challenges, including low academic performance, cognitive impairment, reduced daily functioning and a lack of problem-solving skills. Low academic performance is evident, as the majority of participants have only completed general secondary education. Cognitive impairment and deficiencies in problem-solving skills are closely related to the disease process, as individuals often rely on illicit drugs as a coping mechanism. These challenges negatively impact their resilience and are reflected in the prolonged average delay of 8 years before they decide to initiate treatment. Future interventions should focus on building optimism, strengthening support systems like social connectedness, addressing cognitive challenges, and providing targeted educational and vocational training to mitigate the impact of these adversities.

Our data revealed that a significant percentage of participants began substance consumption during adolescence and spent several years using illicit drugs before seeking treatment. Factors such as family history, temperament, cognitive deficits, executive functioning deficits leading to a lack of self-regulation, poor parenting, and the presence of certain biomarkers, like a diminished P3 component in event-related potentials, have been associated with the early onset of SUD (53). The societal perception of SUDs poses serious barriers for those attempting to access treatment or supportive services. This stigma often results in either reluctance or an inability among individuals with SUDs to seek help, highlighting the critical need for investigations into alternative strategies that can strengthen their resilience against the impacts of their disorders and aid in relapse prevention. One study showed that the transition from substance use to SUD occurs relatively quickly, emphasizing the limited window of opportunity for intervention (54). Targeting adolescents and early detection of SUD through involvement with schools and families appear to be key strategies for enhancing protective factors and reducing adversities. Additionally, incorporating genetic studies could aid in directing early protective interventions; a study found that the *KIAA1211L* locus is significantly associated with OUD among the UAE population (55).

Patients with families having a history of SUD exhibit significantly higher risk of relapse. This finding aligns with previous research, which has shown that inherited genetic factors and learned behaviors significantly contribute to relapse risk (12, 52)​. Therefore, these patients should be a target for healthcare professionals, with extra support and follow-up provided to decrease their risk of relapse. Fostering connectedness, adaptability, spirituality, transcendence, hope, parental monitoring and control, emotional communication, and collaborative problem-solving in affected and vulnerable families (those with poor family relations, poverty, or drug-abusing parents) is crucial for enhancing resilience (56). Furthermore, the media has the power to change the stigmatized view of Emirati patients with SUD. The healthcare system should also be resilient by promptly attending to the needs of patients with SUD. Access to medication, consultations with psychiatrists or psychologists, or admissions should be more accessible than obtaining illegal drugs or seeking advice from other patients with SUD. For example, medications used in the treatment of opioid use disorder (OUD) should be available for patients with comorbidities outside of SUD specialty settings. However, a qualitative study showed that healthcare providers felt that this topic was not clearly addressed in clinical guidelines (57). Providing training for healthcare providers working outside SUD specialty settings, in conjunction with initiating a national online system for the prescription of OUD medications, could enhance the treatment of patients with SUD and prevent misuse.

Participants who are employed demonstrated higher resilience scores and the lowest relapse risk compared to those who were not working. This finding aligns with previous studies (22, 58). Assisting others who are facing stressors may potentially help individuals with SUD mitigate their own adversities and enhance their problem-solving skills. Employment serves as a means of self-actualization, fostering a sense of pride and self-esteem, and providing financial benefits (59). Legislative and policy amendments are essential; many patients are losing their jobs due to incarceration, while others struggle to secure employment because they lack the certificate of good conduct required by employers. Community support, along with proactive policies and healthcare provider involvement, should promote employment support services and instill confidence in the abilities of patients.

Our findings suggest that patients with psychopathology have a higher risk of relapse and lower resilience scores. Patients with co-occurring SUD and psychiatric conditions demonstrate increased adversity, reflected in higher utilization of psychiatric services, greater healthcare costs, severe psychiatric symptoms, elevated suicide risk, and frequent hospital admissions (60, 61); all of which reflect their low resilience and high relapse risk. The adequacy of treatment to patients with SUD comorbid with psychopathology might be one factor in understanding the higher risk of relapse in this group. A study found that the use of anti-SUD medications, such as naltrexone or disulfiram, which have demonstrated some efficacy for treating SUDs, was notably lower among patients with both depression and SUD compared to those with SUD only (62). Others pointed that the lower rates of treatment engagement and adherence in patient with SUD comorbid with PTSD and the variety of approaches used to manage PTSD+SUD, in contrast to the well-established treatment approaches of PTSD and SUD when not comorbid, as factors that could explain the higher risk of relapse (63, 64). However, there is a notable gap in research exploring these dynamics specifically within the UAE context. To address this, further research is needed to investigate access and quality of treatment, the unique socio-cultural, and environmental factors influencing relapse and resilience within this group.

The data indicate that cumulative admission time shows no correlation to resilience, whereas cumulative outpatient (OPD) time does. This difference may be due to the time variable itself, as admission periods tend to be shorter on average, while outpatient periods are considerably longer. To our knowledge, this is the first article that explores the construct of resilience in patients with SUD in the UAE. Introducing interventions for targeting resilience tailored to the UAE context is of great importance. Various interventions support the improvement of resilience, including cognitive-behavioral therapy (65, 66), the five C’s of resilience (confidence/control, connections, commitment, calmness, and care for self), the resilience model, Lazarus’ stress model, stress management, attention interpretation, coping (66), and yoga (67). Additionally, these results support initiating interventions to increase the length of inpatient rehabilitation programs, thereby reducing the incidence of discharge against medical advice. A study showed reduced volumes of gray matter in the prefrontal cortex, which is involved in self-regulation skills, in patients with SUD. This suggests that time is needed to restore normal functioning (68).

Patients using substances such as Valium, Morphine, Heroin, Tramal, Xanax, Lyrica, and Spice have a higher risk of relapse. Moreover, those using more than one substance and those who use a greater number of substances are at an even higher risk of relapse. The “B process” in the context of SUD typically refers to the opponent-process theory, which describes the body’s counter-reaction to a drug’s initial effects. In “Never Enough: The Neuroscience and Experience of Addiction,” Judith Grisel discusses how different substances affect the brain and contribute to the cycle of addiction and why specific substance carries higher risk of relapse than others. For example, when opiates are used, they bind to opioid receptors, producing intense euphoria and pain relief (the A process). In response, the body secretes anti-opiates as a counter-regulatory measure. Notably, this secretion can be triggered not just by the presence of the drug but also by stimuli or environmental cues associated with opiate use. This heightened sensitivity to environmental triggers means that even the anticipation or context of opiate use can induce the secretion of anti-opiates. These anti-opiates help maintain homeostasis but also lead to increased pain sensitivity, dysphoria, dependence, craving, and tolerance during withdrawal (the B process). This cycle, driven by the body’s response to both the drug and its associated cues, significantly contributes to the development, persistence, and higher risk of relapse with opiates compared to other drugs (16). Historically, as the UAE transitioned into a wealthy nation with an enhanced healthcare system, increased access to prescription medications like Xanax, Lyrica, and Valium emerged. This greater availability of these potentially addictive drugs could be contributing to a higher risk of relapse among these substances. Our findings are congruent with the literature emphasizing the higher risk of relapse in polysubstance use disorder, which is also associated with less favorable treatment outcomes and a greater frequency of comorbid mental health conditions, including mood disorders, anxiety disorders, and positive psychotic symptoms (69–71). To mitigate relapse risk, healthcare providers should prioritize careful monitoring and regulation of prescriptions for medications with high abuse potential, such as Xanax, Lyrica, and Valium. Additionally, integrated treatment approaches addressing both substance use and co-occurring mental health conditions may enhance resilience and improve long-term recovery outcomes.

## 6 Limitation

This study employs a cross-sectional design, which is susceptible to the influence of both known and unknown extraneous variables. The absence of statistics regarding the prevalence of SUD among Emirati adult patients hindered the ability to precisely estimate the required sample size. Additionally, a limitation of this study is the inclusion of various types of SUD, potentially introducing confounding variables. However, restricting the study to a single type of SUD would significantly reduce the sample size and the generalizability of the findings.

Another limitation of our study is the potential difference between those who participated and those who did not. We employed a consecutive sampling technique over nine months, including all eligible patients to minimize selection bias and ensure comprehensive representation of Emirati patients. Participation was influenced by factors such as time constraints and concerns about privacy and confidentiality. Notably, as patients attend their appointments periodically, some individuals who initially declined to participate chose to engage in the study during subsequent follow-up appointments, suggesting that nonparticipants may not significantly differ from participants in key characteristics. However, we do not have statistical data to compare participants and nonparticipants directly. Our observations indicate that nonparticipation was mainly due to logistical reasons rather than differing health statuses or demographics. This limitation should be considered when interpreting our findings.

## 7 Conclusion

The research highlights the pressing concern of drug misuse disorders within the UAE, an area that is both stigmatized and under-researched. The study shows that patients diagnosed with SUD in the UAE face a significant risk of relapse, primarily due to their lack of resilience and the various adversities they encounter. These adversities encompass societal stigma, unstructured time, diminished family communication and trust, ongoing associations with drug users, legal issues, employment difficulties, and inadequate treatment, among others.

The correlation between resilience and relapse risk underscores the immediate necessity for customized interventions aimed at strengthening the inherent coping mechanisms of patients with SUD. This study highlights the importance of factors such as societal support, employment, and extended rehabilitation programs that addresses SUD and SUD comorbid with psychopathologies efficiently in enhancing resilience and consequently mitigating the risk of relapse.

Based on the findings of this study, the following recommendations are proposed:

1. Development and implementation of resilience-focused interventions tailored to the UAE context, aiming to enhance resilience in patients with SUD. Techniques such as cognitive-behavioral therapy, stress management, and yoga should be further explored.

2. Strengthening family ties and community support initiatives, including promoting better communication, emotional support, and problem-solving within families, should be encouraged. Similarly, promoting societal understanding and acceptance, possibly through awareness campaigns, can help reduce stigma and encourage social reintegration.

3. Optimizing healthcare systems to adopt a more patient-oriented approach is necessary to enhance the accessibility and convenience of treatments and consultations for patients. Healthcare professionals should receive training to effectively support SUD patients, not only within rehabilitation units but also in primary healthcare settings.

4. Early detection and intervention strategies should be developed for identifying and addressing SUD at a young age, involving families and schools in the process. Promoting employment, volunteering, and charity work opportunities is crucial. Legal and policy amendments are needed to protect the employment rights of individuals with SUD, as employment can significantly enhance self-esteem, financial stability, and overall resilience.

5. Finally, further research is necessary. Future studies should aim to expand our understanding of resilience in the context of SUD in the UAE. Longitudinal research can provide deeper insights into the effectiveness of resilience-based interventions and the long-term outcomes for SUD patients.

## Data Availability

The raw data supporting the conclusions of this article will be made available by the authors, without undue reservation.
